# A 30–60 GHz Broadband Low LO-Drive Down-Conversion Mixer with Active IF Balun in 65 nm CMOS Technology

**DOI:** 10.3390/mi15070845

**Published:** 2024-06-29

**Authors:** Rong Wang, Jincai Wen

**Affiliations:** Key Laboratory of Radio Frequency Circuits and Systems, Ministry of Education, Hangzhou Dianzi University, Hangzhou 310018, China; wr19990324@163.com

**Keywords:** down-conversion mixer, Marchand balun, active balun

## Abstract

A 30~60 GHz broadband down-conversion mixer driven by low local oscillator (LO) power is presented. The down-conversion mixer utilizes an input signal coupling technique based on the Marchand balun to achieve broadband operation and achieves low LO power drive and low DC power consumption through the use of a weak inversion bias with Gilbert switching devices. The broadband conversion of single-ended to differential signals is achieved using the Marchand balun with compensation lines, and an equivalent circuit analysis is performed. For the intermediate frequency (IF) output, a self-biased IF trans-impedance amplifier with current reusing and an active IF balun structure are used to achieve signal amplification and single-ended signal output. Test results show that the proposed mixer achieves a conversion gain of −1.2 to 6.4 dB in an IF output bandwidth of 0.1 to 5 GHz at radio frequency (RF) input frequencies of 30 to 60 GHz and LO driving power of −10 dBm. The DC power consumption of the core mixing unit of the proposed mixer is 4.8 mW, and the DC power consumption including the IF amplifier is 28.3 mW. The proposed mixer uses a 65 nm CMOS technology with a chip area of 0.26 mm^2^.

## 1. Introduction

In recent years, the demand for broadband transceiver systems for high-rate wireless transmission and broadband electromagnetic spectrum monitoring has been increasing. For example, 5th generation wireless communication covers 24.25~52.6 GHz in the millimeter-wave frequency range 2 (FR2) band, which includes the global millimeter-wave unified working frequency bands of 24.25~27.5 GHz and 37~43.5 GHz and regional millimeter-wave frequency bands of 45.5~47 GHz, 47.2~48.2 GHz, and so on. Conventional broadband receivers generally use multiple receivers of different frequency bands in parallel to achieve broadband coverage. Still, the use of multiple circuits, such as mixers, can lead to a significant increase in the size or area of the module or chip, limiting its scale of application. To solve these problems, different frequency bands in the receiver can be covered by a single broadband circuit, avoiding problems such as area and power consumption in conventional broadband receivers.

The broadband down-conversion mixer is the key module in the broadband receiver, and how to realize good performance, including high conversion gain, low noise figure, and low LO driving power operation as well as single-ended output, has become an important attention for high-performance receiver applications. Broadband mixers with a Marchand balun can realize low LO power drive, but their conversion gain is relatively low [1,2]. Among them, the broadband mixer proposed in [1] operates in the range of 20~50 GHz, and the 30~90 GHz broadband mixer proposed in [2] has a conversion gain of only −9.5~−7.5 dB. A distributed mixer based on the 5~45 GHz range has also been proposed, which utilizes a distributed network formed by combining MOS transistors with transmission line structures. This allows for the circuit to maintain uniform gain and phase response over a wide frequency range, enabling broadband operation with only 1.4 mW of DC power consumption to achieve a 10 dB conversion gain. However, due to the use of a large number of transmission lines, the circuit area is also large, and high LO driving power is required [3]. The output of the double-balanced mixer is usually in differential form, which can be realized by using an active balun or buffer circuit at the output port to provide additional gain while providing a single-ended output, but it requires high LO power to drive and increases the power consumption of the circuit design [4,5,6,7]. How to realize a broadband down-conversion mixer with low power consumption, low LO power drive, and a certain conversion gain is the difficulty of this design.

Aiming at the challenges of broadband monolithic circuits in millimeter-wave bands as well as low-signal source driving, this article realizes a 30~60 GHz broadband low-power down-conversion mixer based on a 65 nm CMOS technology by selecting appropriate device operating states and passive matching structures as well as using an active IF balun to realize single-ended signal output, which facilitates the extended use in a broadband receiver system.

## 2. Circuit Design

### 2.1. Design of Mixer

**Figure 1:**
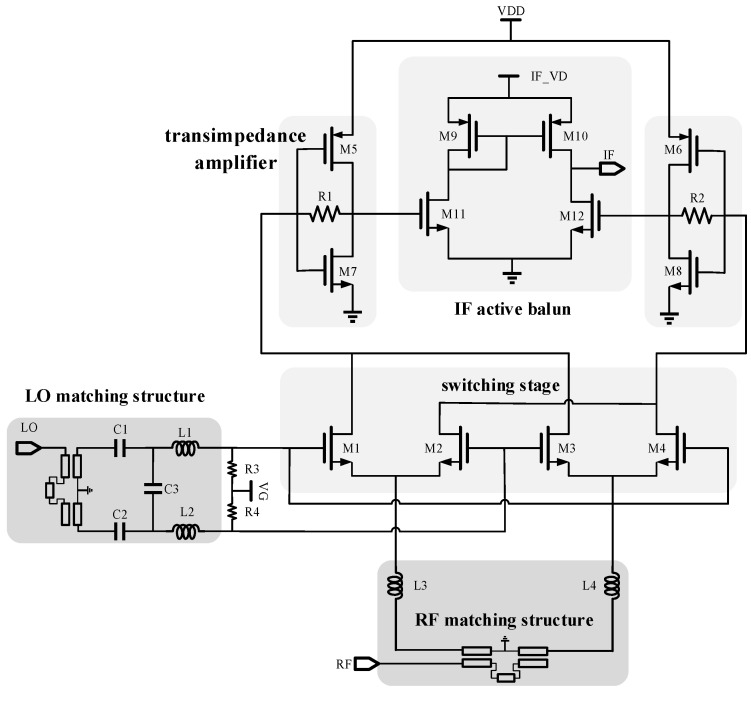
The structure of the down-conversion mixer.

The down-conversion mixer structure is shown in Figure 1, which mainly consists of the switching stage, matching structure, IF transimpedance amplifier, and IF active balun. The switching stage utilizes a Gilbert cell designed to achieve high port isolation, simultaneously enabling differential input and output [8]. The matching stage uses an on-chip Marchand balun to realize the conversion of single-ended signals to differential signals with broadband impedance matching. In order to improve the conversion gain of the mixer, a trans-impedance amplifier that reuses the current from the switching stage is employed, and it also achieves the conversion of the output current signal of the switching stage into a voltage signal. The IF active balun is a differential amplifier with current mirrors acting as loads to realize the conversion of differential signals to single-ended signal outputs and provide additional gain.

**Figure 2:**
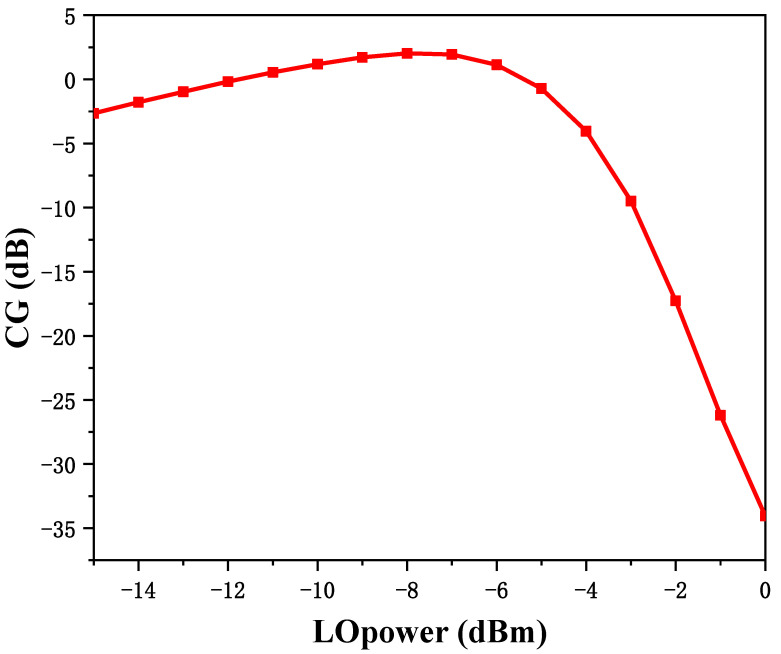
The characteristic of conversion gain with LO drive power.

The conventional Gilbert switching transistor operates in the strong inverse bias region (*V*_GS_ > *V*_TH_; *V*_GS_ is the MOS gate-source voltage, and *V*_TH_ is the MOS threshold voltage), but when the switching transistor operates in the weak inverse bias region (*V*_GS_ > *V*_TH_), the NMOS transistors drain current *I*_D_ and exhibit an exponential function relationship with *V*_GS_ instead of a square law characteristic, and the NMOS transistors operating in this state have a higher current ratio; at this time, the conversion gain (CG) of the mixer is shown in Equation (1) [9]:

(1) $$CG_{p}=\frac{g_{m}^{2}}{4n^{2}V_{T}^{2}}V_{\mathrm{LO}}^{2}R_{\mathrm{RF}}R_{\mathrm{IF}}$$

where *CG*_P_ is the power gain, *V*_LO_ is the LO signal amplitude, *g*_m_ is the transconductance of the transistor, *R*_RF_ and *R*_IF_ are the loads at the IF and RF ports, respectively, *V*_T_ (≈25 mV) is the thermal voltage, and n (1~3) is the slope factor of the weakly inverting type. According to this equation, it can be observed that, at low LO driving power, it is insensitive to LO power compared with the strong inverse type; therefore, to realize the condition of equal conversion gain, relatively low LO power is needed compared with the strong inverse type. Thus, the gate bias of the switching stage is finally chosen to be 0.3 V, as shown in Figure 2, which can reduce the requirement for LO driving power.

The gate lengths of the switching transistors (M1~M4) are set to 60 nm. The choice of gate width affects the gate-source parasitic capacitance (*C*_GS_). The wider the gate, the larger the *C*_GS_, which results in a smaller imaginary part of the input impedance and does not increase the difficulty of matching. After considering both conversion gain and matching difficulty, the gate width of 20 um is selected.

The load of the mixer uses a trans-impedance amplifier with resistive feedback, which also realizes the conversion of the output current signal to a voltage signal compared to the traditional resistive load but can provide additional gain. The PMOS (M5) is connected across the NMOS (M7), which makes the circuit trans-conductance change from the original *g*_m7_ to *g*_m5_ + *g*_m7_, which increases the trans-conductance of the circuit, the gain is A_V_ = −(*g*_m5_ + *g*_m7_)(*r*_o5_//*r*_o7_), and the output impedance is *r*_o5_//*r*_o7_. However, this configuration requires an additional gate bias voltage. Thus, the resistor (R1) at the gate and the drain of NMOS (M7) provide gate bias for this trans-impedance amplifier without requiring an extra DC power supply. Meanwhile, the current flowing out of the PMOS (M5) flows through the feedback resistor (R1) to the switching stage NMOS (M1, M3) to realize current reuse, which can reduce DC power consumption. According to the calculation of the trans-impedance of this structure in [10], the size of the MOS transistors and the resistance value of the feedback resistor together determine the gain of this trans-impedance amplifier.

Finally, considering the trade-off between conversion gain and DC power consumption, the size of the PMOS (M5, M6) is 48 μm/60 nm (gate width/gate length), the size of the NMOS (M7, M8) is 40 μm/60 nm, and the resistance value of the feedback resistors (R1, R2) is 800 Ω. The trans-impedance amplifier, together with the switching stage, has a DC power consumption of 4.8 mW.

### 2.2. Broadband Balun Design

The input of the mixer is a single-ended signal, but the switching stage is a differential signal input, so the matching network must not only achieve broadband matching but also complete the conversion of single-ended signals to differential signals. This conversion process is usually realized using baluns, which are classified into active and passive baluns. Active baluns can provide additional gain but also induce additional DC power consumption. Passive baluns can be further classified into lumped-element baluns, spiral transformer baluns, and distributed-parameter baluns [11]. In this design, the Marchand balun is selected, which not only has good amplitude-phase balance performance but also has excellent broadband operation [12].

**Figure 3:**
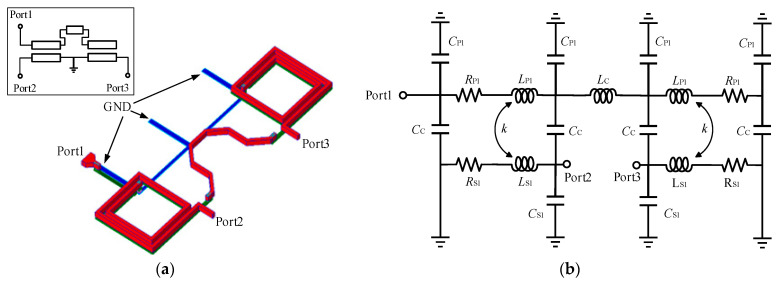
The 3D structure (**a**); simplified equivalent circuit model (**b**) of the Marchand balun.

The 3D structure of the broadband balun is shown in Figure 3a. The primary and secondary coils of the balun use the top two layers (M9 and M8) of metal, respectively. The resistivity of the M9 layer is 5 mΩ/sq, and the resistivity of the M8 layer is 21 mΩ/sq, which is much smaller than the resistivity of the other metal layers (e.g., metal M1~M7) of 160 mΩ/sq, which can reduce the signal loss of the balun. Furthermore, the introduced compensation line can improve the amplitude-phase balance performance. Meanwhile, the size of the balun can be reduced by further folding the compensation line, achieving a trade-off between performance and area.

**Figure 4:**
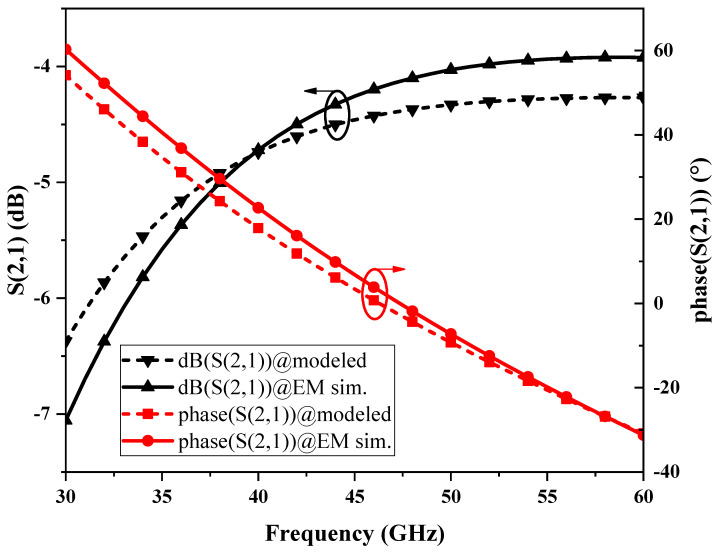
Results of fitting the balun Port2 equivalent circuit model to the actual layout.

Based on the structure of the Marchand balun, its simplified equivalent circuit model can be proposed as shown in Figure 3b, where *L*_P1_ and *L*_S1_ are the self-inductance of the primary and secondary coils, *k* is the coupling coefficient between the primary and secondary coils, *R*_P1_ and *R*_S1_ are the parasitic resistances of the primary and secondary coils, *C*_C_ is the coupling capacitance between the primary and secondary coils, *C*_P1_ and *C*_S1_ are the parasitic capacitances between the coils and the substrate, and *L*_C_ is the inductance of the compensating line; the values of the parameters of each device are shown in Table 1. The main parameters of the Marchand balun used in the matching network include a line width of 2 μm, a line gap of 2 μm, and an outer width of the coil of 42 μm, and the number of coils is 1.75 turns. The equivalent circuit model of the Marchand balun is compared with the electromagnetic (EM) simulation results, as shown in Figure 4, and the model can fit the EM simulation results well.

**Figure 5:**
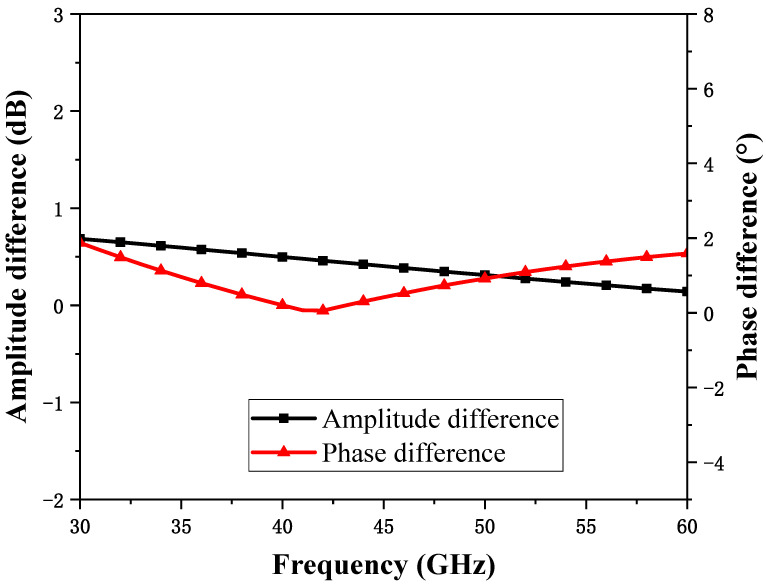
Balun amplitude and phase balance performance.

The broadband baluns used at the RF and LO ports have the same sizes, and their amplitude-phase balance characteristics in the operating frequency band are shown in Figure 5. It can be observed that, in the frequency range of 30~60 GHz, the amplitude imbalance of the balun is less than 0.7 dB, and the phase imbalance is less than 2°. The simulation result demonstrates that the balun structure has a good amplitude-phase balance characteristic under a wide frequency band and has a small size, with an area of only 155 μm × 42 μm.

**Figure 6:**
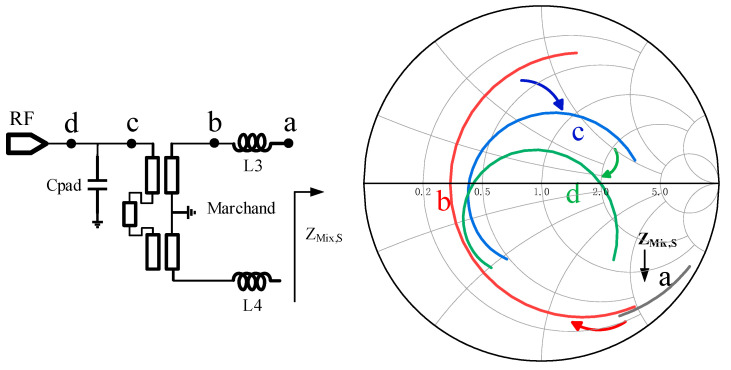
RF port matching structure and impedance conversion path.

While designing the Marchand balun structure, it is also necessary to comprehensively consider the impedance matching design with the mixing core unit. Taking the RF port matching network as an example, a matching network composed of a series inductor, Marchand balun, and capacitor can be used, and its matching structure and matching conversion path are shown in Figure 6. Among them, the inductor *L*_3_ has 1.5 turns and a 31.5 μm inner diameter, with a line width and spacing of 3 μm and 2 μm, respectively, and its inductance is approximately 170 pH. The *C*_pad_ is the PAD parasitic capacitor with a capacitance of 27 fF. It can be found that the matching network can realize the matching design in the broadband operating range from 30 to 60 GHz, and it is characterized by a simple structure and compact area.

### 2.3. IF Active Balun

**Figure 7:**
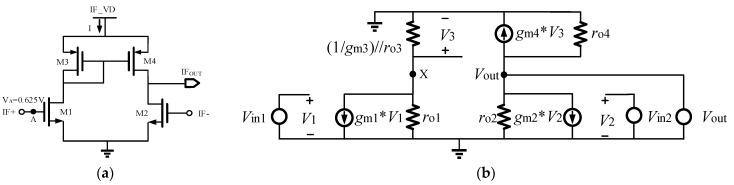
Active IF balun schematic (**a**) and small signal model (**b**).

The IF signal output by the trans-impedance amplifier is a differential signal, usually using the transformer balun to realize the differential signal into a single-ended signal output but at relatively low IF output frequencies, such as below GHz; employing passive balun matching can significantly increase its area or even render its realization challenging. Here, the active IF balun is used and shown in Figure 7, which is a differential amplifier with an active current mirror acting as a load, not only realizing a single-ended signal output but also providing an additional IF gain, with an area of only 87 × 26 μm^2^.

The equivalent small-signal model of the circuit is shown in Figure 7. *g*_m1_, *g*_m2_, and *g*_m4_ are the trans-conductance of the M1, M2, and M4 transistors, respectively, and *r*_o1_, *r*_o2_, and *r*_o4_ are the channel resistor of the M1, M2, and M4 transistors, respectively, with *r*_d_ = (1/*g*_m1_)//*r*_o1_. The small-signal model of the M3 transistor can be equivalent to the resistance value of (1/*g*_m3_)//*r*_o3_ (*g*_m3_ is the trans-conductance of M3, and *r*_o3_ is the channel resistance of the M3 transistor). This can be obtained from Figure 7:

(2) $$g_{m1}V_{1}+\frac{V_{3}}{r_{o1}}+\frac{V_{3}}{r_{d}}=0$$

(3) $$g_{m2}V_{2}+\frac{V_{\mathrm{out}}}{r_{o2}}-\frac{V_{3}}{r_{d}}=0$$

Since *V*_1_ − *V*_2_ = *V*_in1_ − *V*_in2_, *g*_m1_ = *g*_m2_ = *g*_mN_, and *g*_m3_ = *g*_m4_ = *g*_mP_, the two equations are subtracted to obtain the following:

(4) $$g_{\mathrm{mN}}\left(V_{\mathrm{in}1}-V_{\mathrm{in}2}\right)+\frac{V_{3}}{r_{\mathrm{oN}}}-\frac{V_{\mathrm{out}}}{r_{\mathrm{oN}}}+\frac{2V_{3}}{r_{d}}=0$$

(5) $$A_{v}=\frac{V_{\mathrm{out}}}{V_{\mathrm{in}1}-V_{\mathrm{in}2}}=g_{\mathrm{mN}}\frac{2r_{\mathrm{oN}}r_{\mathrm{oP}}g_{\mathrm{mP}}}{1+2r_{\mathrm{oP}}g_{\mathrm{mP}}+2r_{\mathrm{oP}}g_{\mathrm{mP}}}$$

**Figure 8:**
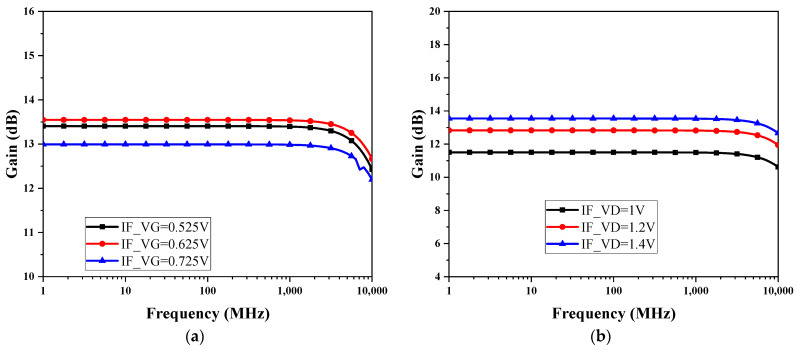
The characteristic in active IF balun gain with IF_VG (**a**) and IF_VD (**b**).

**Figure 9:**
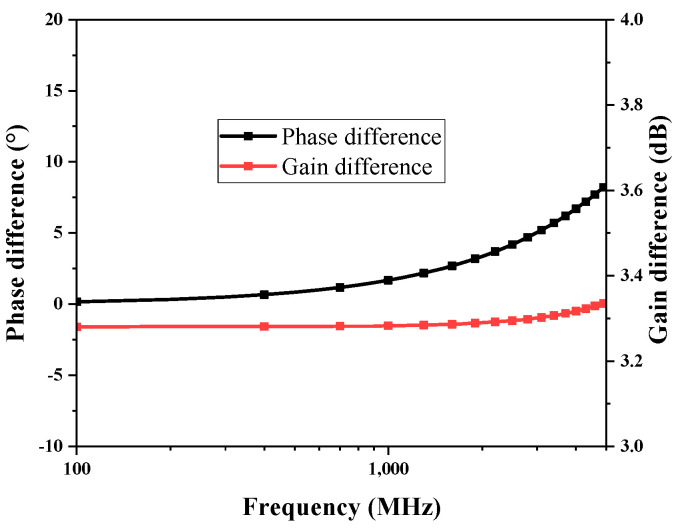
Active balun gain and phase balance performance.

When 2*r*_oP_*g*_mP_ + 2*r*_oP_*g*_mP_ >> 1, the voltage gain of this circuit can be approximated as *A*_V_ = *g*_mN_(*r*_oP_//*r*_oN_); therefore, it is necessary to rationally design the trans-conductance of the M1 and M2 transistors to obtain a suitable gain. Since the diode-connected PMOS device consumes a certain voltage margin, the circuit needs to be at a higher voltage, but the higher the voltage, the higher the power consumption; therefore, a compromise is needed. Figure 8a shows the gain of this active IF balun at different gate voltages; it can be observed that the output DC voltage of the front-connected trans-impedance amplifier 0.625 V is a more appropriate input gate bias for this active IF balun, and ±0.1 V change, there is only a gain drop of 0.55 dB. Figure 8b shows the gain of this active IF balun at different drain voltages; the gain is considered in trade-off with the power consumption, take IF_VD = 1.4 V, and the DC power consumption is 23.5 mW. The gain variation caused by bias conditions can be compensated in other circuits of the receiver system to achieve stable gain amplification. Due to the asymmetry of the structure of this active balun circuit, the gain and phase balance performance is slightly weak, as shown in Figure 9. In the frequency range of 0.1 to 1 GHz, the phase imbalance is 1.6°, and the gain imbalance is approximately 3.3 dB.

## 3. Measurement Results

**Figure 10:**
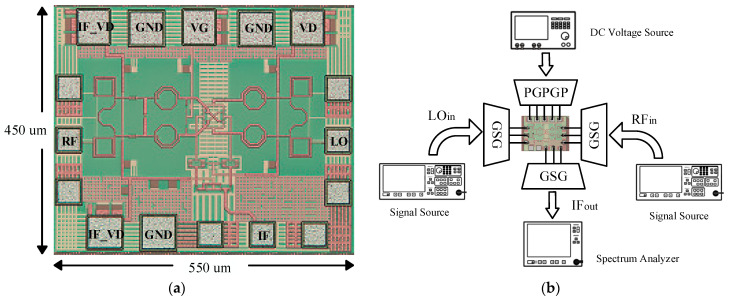
Down-conversion mixer chip photo (**a**) and test scheme (**b**).

The down-conversion mixer is designed in a 65 nm CMOS technology with the total area of 0.55 × 0.45 mm^2^, and the chip photo is shown in Figure 10a. The mixer is tested using chip-on-board, and the test scheme is shown in Figure 10b. Among them, the RF and the LO use an Agilent E8257D signal source and a Ceyear 1465L signal source, respectively, to provide millimeter-wave signals, and the output is tested using an Agilent E4440A Spectrum Analyzer. The RF, LO, and IF use ground-signal-ground (GSG) probes to input or output the signals, and the bias conditions given are VG = 0.3 V, VD = 1 V, and IF_VD = 1.4 V.

**Figure 11:**
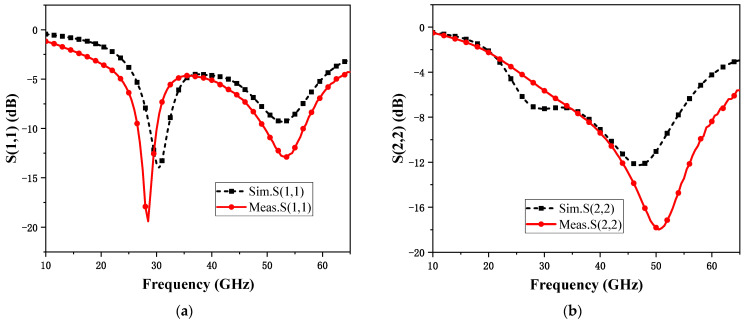
Mixer RF (**a**) and LO (**b**) port S-parameter test and simulation results.

**Figure 12:**
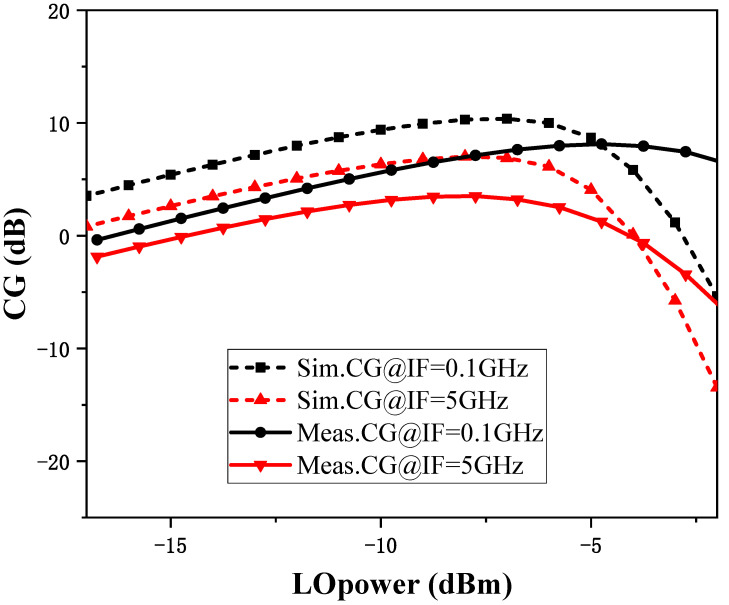
Simulation and test results of conversion gain with LO power.

Figure 11 shows the S-parameter comparison of the measurement and simulation between the RF and LO ports of the mixer, and it can be observed that these two results have good consistency. When testing frequency conversion characteristics, it is necessary to first determine the appropriate LO driving power. Figure 12 shows the simulation and test results comparing the conversion gain with LO input power for a fixed RF frequency of 45 GHz and IF frequencies of 0.1 GHz and 5 GHz, from which it can be observed that the optimal LO input power is below −5 dBm, which reduces the requirement for LO driving power compared with other designs. Secondly, the difference in the best LO drive power between the simulation results and the test results may be due to the fact that the accuracy of the MOS transistor nonlinear modeling affects parameters such as the MOS transistor *V*_TH_ when the switching stage may not accurately operate in the weak inverse bias region, which affects the mixer’s conversion gain as well as the requirement for the LO drive power. In subsequent testing, the LO driving power will consistently be set at −10 dBm.

**Figure 13:**
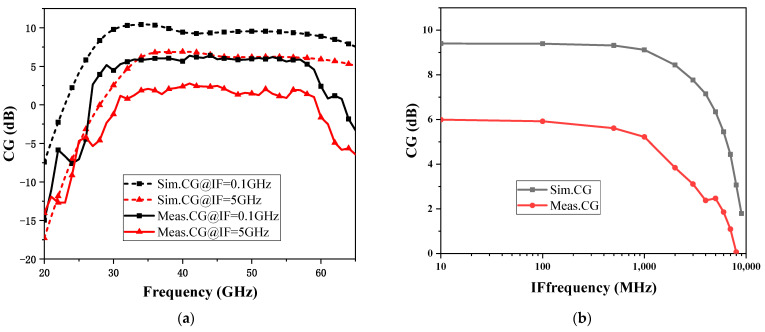
Simulation and test results of conversion gain with RF (**a**) and IF frequency (**b**).

Conversion gain is an important indicator of mixer performance. Conversion gain typically needs to represent as two different frequency characteristics, namely RF input frequency and IF output frequency. Figure 13a shows the comparison of the simulation and test results of conversion gain with RF input frequency for fixed IF frequencies of 0.1 GHz and 5 GHz. The conversion gains are 2.4~6.4 dB (IF frequency of 0.1 GHz) and −1.2~2.45 dB (IF frequency of 5 GHz) in the 30~60 GHz, respectively. Similar to the power characteristics shown in Figure 12 at fixed frequency, due to the inaccuracy of the MOS nonlinear model in the millimeter wave band, the difference between the test and the simulation results on the broadband frequencies is approximately 5 dB. Figure 13b shows the simulation and test comparison of the conversion gain with IF frequency at a fixed RF frequency of 45 GHz, and it can be observed that the conversion gain stabilizes at 5~6 dB at an IF frequency of 0.01~1 GHz and gradually decreases at an IF frequency of 1~10 GHz.

**Figure 14:**
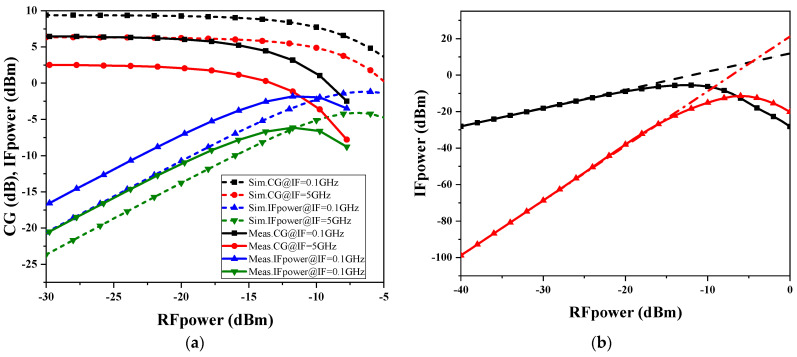
Down-conversion mixer linearity performance (**a**) IP1dB and (**b**) IIP3.

The mixer is an important module in the receiver, and its linearity will limit the dynamic range of the entire receiver; here, we use the input 1dB compression point as well as the input of the third-order intermodulation point to measure the linearity of this design. Figure 14a shows the simulation and test results of the conversion gain and IF output power variation with RF input power when the RF frequency is 45 GHz, and it can be observed that the IP1dB compression point of the mixer is approximately −16 dBm. Due to the fact that the third-order intermodulation point needs to be used with multiple millimeter-wave band sources, only the simulation results of the IIP3 are given. The third-order intermodulation point simulation results for this mixer are shown in Figure 14b, with a fixed RF frequency of 45 GHz, an IF frequency of 100 MHz, and an IP3 of approximately −4.6 dBm.

**Figure 15:**
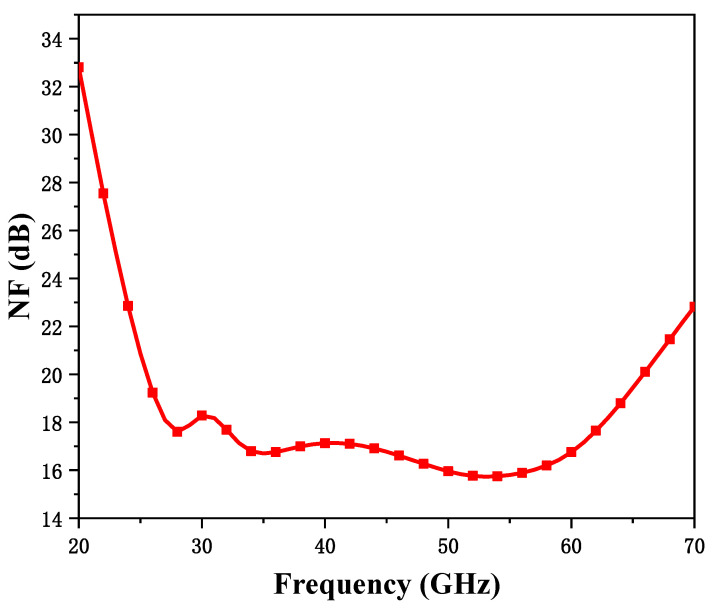
Noise finger simulation results.

The simulation results of the noise figure of the mixer are shown in Figure 15, with a fixed RF frequency of 45 GHz, an IF frequency of 100 MHz, and a noise figure of 15.7 to 18.2 dB. This noise figure is not very excellent, but a low-noise amplifier will be front-loaded to improve the noise performance of the entire receiver system.

Table 2 shows a detailed performance comparison between the mixer design and recent works. The proposed down-conversion mixer ensures a certain conversion gain at a low LO driving power while achieving a wide RF bandwidth, and it has the smallest chip area among the compared designs.

This article proposes a 30~60 GHz broadband low LO drive down-conversion mixer with an active IF balun, which was fabricated using a 65 nm CMOS technology. The IF output uses a resistive feedback trans-impedance amplifier as well as an IF active balun, which not only realizes the suitable IF gain but also obtains the conversion of differential signals to single-ended signals. According to the test results, the down-conversion mixer has an IF bandwidth of 0.1 to 5 GHz and can achieve a conversion gain of −1.2 to 6.4 dB at an RF frequency of 30 to 60 GHz and an LO drive power of −10 dBm. The down-conversion mixer has good advantages in terms of operating bandwidth, conversion gain, and chip area and has low requirements for the LO driving power, which makes it suitable for applications in broadband millimeter-wave receiver circuits.

## 4. Conclusions

This article proposes a 30~60 GHz broadband low LO drive down-conversion mixer with an active IF balun, which was fabricated using a 65 nm CMOS technology. The IF output uses a resistive feedback trans-impedance amplifier as well as an IF active balun, which not only realizes the suitable IF gain but also obtains the conversion of differential signals to single-ended signals. According to the test results, the down-conversion mixer has an IF bandwidth of 0.1 to 5 GHz and can achieve a conversion gain of −1.2 to 6.4 dB at an RF frequency of 30 to 60 GHz and an LO drive power of −10 dBm. The down-conversion mixer has good advantages in terms of operating bandwidth, conversion gain, and chip area and has low requirements for the LO driving power, which makes it suitable for applications in broadband millimeter-wave receiver circuits.

## Figures and Tables

**Table 1:** Marchand balun equivalent circuit parameters.

*L*_P1_	*L*_S1_	*L*_c_	*k*	*R*_P1_
245 pH	207 pH	61 pH	0.83	4.6 Ω
*R*_S1_	*C*_C_	*C*_P1_	*C*_S1_	
1.2 Ω	18 fF	9 fF	7.5 fF	

**Table 2:** Comparison of broadband down-conversion mixer performance.

Ref.	Technology	RF Bandwidth/GHz	IF Bandwidth/GHz	P_LO_/dBm	CG/dB	NF/dB	Power/mW	Output	Area/mm^2^
[1]	90 nm CMOS	20~50	2.6	0	−2~2	16.7~17.7	6	Differential	0.48
[2]	90 nm CMOS	30~90	-	2.3~2.6 ^①^	−9.5~−7.5 ^①^	-	0.6	Differential	0.39
[3]	180 nm CMOS	5~45	0.5~5.5	8	11~13.2	7.6~10.2	1.4	Single-ended	0.66
[4]	0.25 μm GaAs HEMT	7~42	DC~4	0~13	−6~6	-	-	Single-ended	2.55
[5]	40 nm CMOS	24−40	8	−10	−4.1~1.2	12.4~15.4	28.3	Single-ended	0.65
[6]	65 nm CMOS	26~39	-	5	4.8~6.5	12.5~13.5	10.3	Single-ended	0.4
[7]	90 nm CMOS	25~45	0.5~12	4	−5.5~−8.5	12.4~20	-	Single-ended	0.4
This work	65 nm CMOS	30~60	0.1~5	−10	−1.2~6.4	15.7~18.2 ^②^	4.8/28.3	Single-ended	0.25

^①^: At an LO frequency of 40 GHz. ^②^: Simulation results of mixer with IF active balun.

## Data Availability

Data are contained within the article.
